# Clinical validation of a spectroscopic liquid biopsy for earlier detection of brain cancer

**DOI:** 10.1093/noajnl/vdac024

**Published:** 2022-02-22

**Authors:** James M Cameron, Paul M Brennan, Georgios Antoniou, Holly J Butler, Loren Christie, Justin J A Conn, Tom Curran, Ewan Gray, Mark G Hegarty, Michael D Jenkinson, Daniel Orringer, David S Palmer, Alexandra Sala, Benjamin R Smith, Matthew J Baker

**Affiliations:** 1 Dxcover Ltd. (formerly known as ClinSpec Diagnostics Ltd.), Glasgow, UK; 2 Translational Neurosurgery, Centre for Clinical Brain Sciences, University of Edinburgh, Edinburgh, UK; 3 Children’s Mercy Research Institute at the Children’s Mercy Hospital, Kansas City, Kansas, USA; 4 Independent Health Economics Consultant, Edinburgh, UK; 5 Institute of Translational Medicine, University of Liverpool & The Walton Centre NHS Foundation Trust, Liverpool, UK; 6 Department of Neurosurgery, New York University Grossman School of Medicine, New York, New York, USA; 7 Department of Pure and Applied Chemistry, University of Strathclyde, Glasgow, UK

**Keywords:** brain cancer, clinical spectroscopy, earlier detection, infrared, liquid biopsy

## Abstract

**Background:**

Diagnostic delays impact the quality of life and survival of patients with brain tumors. Earlier and expeditious diagnoses in these patients are crucial to reduce the morbidities and mortalities associated with brain tumors. A simple, rapid blood test that can be administered easily in a primary care setting to efficiently identify symptomatic patients who are most likely to have a brain tumor would enable quicker referral to brain imaging for those who need it most.

**Methods:**

Blood serum samples from 603 patients were prospectively collected and analyzed. Patients either had non-specific symptoms that could be indicative of a brain tumor on presentation to the Emergency Department, or a new brain tumor diagnosis and referral to the neurosurgical unit, NHS Lothian, Scotland. Patient blood serum samples were analyzed using the Dxcover® Brain Cancer liquid biopsy. This technology utilizes infrared spectroscopy combined with a diagnostic algorithm to predict the presence of intracranial disease.

**Results:**

Our liquid biopsy approach reported an area under the receiver operating characteristic curve of 0.8. The sensitivity-tuned model achieves a 96% sensitivity with 45% specificity (NPV 99.3%) and identified 100% of glioblastoma multiforme patients. When tuned for a higher specificity, the model yields a sensitivity of 47% with 90% specificity (PPV 28.4%).

**Conclusions:**

This simple, non-invasive blood test facilitates the triage and radiographic diagnosis of brain tumor patients while providing reassurance to healthy patients. Minimizing time to diagnosis would facilitate the identification of brain tumor patients at an earlier stage, enabling more effective, less morbid surgical and adjuvant care.

**Key Points**

- This prospective, analyst-blinded clinical study demonstrates the clinical utility of a spectroscopic brain cancer liquid biopsy which can effectively triage symptomatic patients for investigative medical imaging for suspected brain tumors.

**Importance of the Study**

This prospective, analyst-blinded clinical study demonstrates the clinical utility of a spectroscopic brain cancer liquid biopsy which can effectively triage symptomatic patients for investigative medical imaging for suspected brain tumors. A simple, rapid blood test that can be administered easily in a primary care setting would enable quicker referral to imaging for those who are most likely to have a brain tumor. Here we present our results from machine learning models that have been tuned for greater sensitivity or specificity, since the precise trade-off between these statistics is likely to vary across international healthcare systems. Our findings indicate the Dxcover® Brain Cancer liquid biopsy has comparable performance to commercially available tests for other cancers.

This year in the United States, it is estimated that over 80 000 people will likely be diagnosed with a brain or central nervous system tumor.^1^ Although progress has been made in the classification of these tumors, which comprise over a hundred distinct types based on histopathologic criteria and immunohistochemical data set forth by the World Health Organization, there is a very high fatality rate with only one-third of patients surviving five years or more after a diagnosis.^2^ This is likely due to diagnostic delays as early symptoms of brain cancer are often nonspecific; for example, headache is the most common symptom, hence there is an excessive number of brain scans undertaken to identify a tumor. Early detection is therefore challenging because of the range of disorders that these indicators can be linked to.^3,4^ Delayed detection leads to 62% of brain tumor patients receiving a diagnosis when they present to the emergency department (ED) with advanced symptoms like seizures and neurological deficits.^5^ Many of these patients will have previously visited their primary care doctor on several occasions with non-specific symptoms, and since brain tumors are rare, a non-tumor diagnosis is much more likely. Consequently, brain tumor patients see their primary care physician on average three times before a final diagnosis.^6^ Early referral for brain imaging is crucial, but existing symptom-based referral guidelines inadequately stratify patients for brain imaging based on suspicion of cancer which may only be made after a lengthy process of eliminating other conditions that are a part of differential diagnosis. The National Institute for Health and Care Excellence (NICE) provides referral guidance in the UK, while the not-for-profit organization National Comprehensive Cancer Network (NCCN) provides this guidance in the USA. The maximum positive predictive value (PPV) of NICE symptom-based referral guidelines for suspected brain cancer, for the presence of “symptoms related to the central nervous system,” is only 2.9%.^7^ New rapid and affordable tests are required to support clinical decision-making, with improved diagnostic sensitivity and specificity so that at-risk patients do not fall through the cracks of the healthcare system.

Liquid biopsy technologies, primarily based on blood testing, have the potential to transform cancer diagnostics.^8,9^ Most liquid biopsies focus on a genomic approach where genetic material, such as circulating tumor DNA (ctDNA), is measured and quantified.^10^ This method relies upon the target ctDNA being released by the tumor and navigating its way into the bloodstream, followed by amplification for detection. The amount of cancerous genetic material available for analysis is minute, and since mutations in ctDNA can also be found in healthy individuals, it is very difficult to diagnose cancer with this strategy. In fact, when the fraction of DNA is below only 1 cancer genome to 10 000 normal genomes, even with 10 mL of patient blood it is possible that there would be no single genome available for sequencing.^11^ Additionally, ctDNA is only found in 75% of patients with metastatic cancer.^12^ Importantly, not all tumors or tumor sub-types appear to release ctDNA into the bloodstream, and some cancers are known for their low ctDNA concentration (such as brain or prostate cancer) adding to the poor reliability of genetic markers, particularly for early-stage cancers which shed such a low amount of ctDNA that current techniques are incapable of detecting them.^13–15^

In brain cancer, a liquid biopsy could rapidly triage the symptomatic population in a primary care setting with a possible brain tumor diagnosis. The most at-risk patients could then be fast-tracked for urgent brain imaging. A spectroscopic liquid biopsy is unique in that it produces a biological signature, representing the whole biochemical profile of the serum sample and containing molecular information from the host response as well as the tumor (Supplementary Figure S1.).^16^ In a spectroscopic approach the full range of biomarkers within blood serum is interrogated, rather than being limited to the analysis of individual molecular classes, such as ctDNA. We have recently demonstrated that, unlike other liquid biopsies, the test performance is insensitive to tumor size—down to as small as 0.2 cm^3^—illustrating great promise for the deployment of the technique for early detection and diagnosis.^17^

There are currently no available tests tailored for the early detection and triage of brain cancer, yet there are several that target other types of cancer. These tests focus on the analysis of a symptomatic patient population and the results of the test are used to triage the patient towards medical imaging or back to the primary care clinician (Figure 1). The SelectMDx test for prostate cancer, for example, is a urine-based test that has been successfully launched in the USA and Europe by MDxHealth.^18^ Likewise, the ExoDx test is an exosome-based urine test that provides an individual risk score for clinically significant prostate cancer.^19^ Further examples include the Progensa PCA3 prostate cancer test from HOLOGIC,^20^ Cologuard (Exact Sciences), and Colox (Novigenix) tests for colorectal cancers,^21,22^ and the EarlyCDT test (Oncimmune) for lung cancer.^23^ The use of these tests supports the clinical utility of triage strategies within healthcare systems. A simple, rapid blood test to support clinical decision-making in ruling out a possible brain tumor in symptomatic patients would facilitate faster diagnosis. Earlier diagnosis, when tumors are smaller, can expedite access to life-saving treatments, increase the success of these interventions, and reduce harm from more invasive surgeries and aggressive therapies. The novel Dxcover® Brain Cancer liquid biopsy uses infrared (IR) light to interrogate patient blood sera. Indicators of disease are present within the generated spectroscopic signature and are accentuated and classified by a diagnostic machine learning algorithm. The results of the liquid biopsy will facilitate clinical decision-making to determine the appropriate referral path for at-risk patients. A positive result would indicate an increased risk for brain cancer and the patient would be expedited to imaging to receive a diagnosis much earlier than if they had not received the liquid biopsy test. A negative result would inform the patient and provider that there is not an increased risk of brain cancer and the patient would receive care in accordance with routine follow-up protocols and procedures based on clinical judgment. Patients, for whom the predicted risk of cancer is low, could avoid the potential harm from brain imaging in terms of radiation and the identification of incidental abnormalities prompting further investigations or treatments. Furthermore, the potential for cost savings has also been demonstrated when considering the reduction in unnecessary brain scans, as well as reducing the burden on hospitals and clinicians, and other preventable tests or procedures.^24,25^

**Figure 1.**
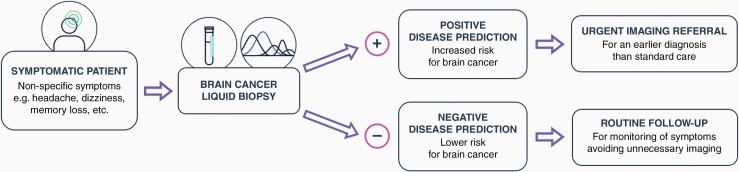
Patient pathway with the addition of a brain cancer liquid biopsy for triage.

Over the past decade, the differentiation between brain cancer and non-cancer has been investigated using this approach, first utilizing retrospectively collected serum samples from biobank facilities.^26–31^ Moreover, we recently illustrated the potential to distinguish between various brain tumor subtypes.^32^ To explore the true clinical utility, the first prospective study in a targeted medical environment was conducted using the spectroscopic liquid biopsy from patients referred by their primary care doctor for urgent brain imaging.^33,34^ This landmark study achieved 92% sensitivity for the most common brain tumor—glioblastoma multiforme (GBM)—and 81% sensitivity for all brain tumors, with 80% specificity. We now present data on samples collected from 603 patients in a more challenging cohort of symptomatic patients, which includes the presence of other serious medical conditions presenting in the emergency department, such as stroke. This accurately represents the target population and consideration of these other pathologies is key for patient triage, as these pathologies will statistically be the more likely ailment when patients present with non-specific symptoms. We report the clinical utility of the brain cancer blood test within this cohort and assess how the diagnostic models can be tailored towards higher sensitivity or specificity depending on clinical priorities.

## Materials and Methods

### Patient Sample Selection

Ethical approval for this study was granted by Lothian REC (15/ES/0094). Blood samples were gathered at three different collection sites, from patients representing a diverse, urban patient population and aged 16 years or older were eligible for inclusion, encompassing different points on the cancer referral and diagnosis pathway.

1. Patients referred by their primary care doctors in Lothian (UK) who have direct access CT (DACT) brain imaging for exclusion of significant intracranial pathology, where urgent (same day) brain imaging is not required. All DACT brain imaging is performed in a single neuroradiological department at the Department of Clinical Neurosciences, Western General Hospital, Edinburgh, Scotland. Patients referred for DACT or within the brain cancer population, who were able to provide informed consent, were invited to participate.
2. Patients presenting to the Emergency Department of the Royal Infirmary of Edinburgh with a new severe onset neurological symptom—eg, seizures, fainting, memory loss, vision change etc.—with no history of trauma, where the assessing clinician judged brain imaging was necessary and that the differential diagnosis included a brain tumor. Patients aged 16 years or older, who were able to provide informed consent were invited to participate.
3. Patients with a new brain tumor diagnosis, scheduled for surgery at the Western General Hospital, Edinburgh, NHS Lothian, were also invited to participate prior to surgery.

Investigators obtained written informed consent from all study participants. In DACT patients a blood sample was collected via venepuncture during their attendance for brain imaging appointments. In collection sites 2 and 3, a blood sample was taken during a clinical assessment.

Blood samples were obtained during routine venepuncture using S-Monovette 7.5 ml Serum Gel Z collection tubes (Sarstedt, Germany) and anonymized for processing and analysis. Each sample was gently inverted 8 times and allowed to clot. Centrifugation was performed for 15 minutes at 2200 g (or equivalent) and stored in a –80°C freezer. Data interpretation was blinded to brain imaging and histological diagnosis; imaging outcomes were recorded from the formal radiological report and histological tumor diagnosis was available for patients that underwent surgery. Clinical data were entered into an online database designed and supported by Edinburgh Clinical Trials Unit, which meets standards on data security and participant privacy and confidentiality.

### Patient Sample Analysis

Patient serum samples were analyzed using the Dxcover® Brain Cancer liquid biopsy. For further details, we direct the reader to the following articles.^33,34^ In this study, patient whole blood samples were collected and processed on-site via standard clinical sample preparation procedures prior to brain imaging or brain tumor surgery at the Western General Hospital, Edinburgh, NHS Lothian. The resultant serum samples were stored at –80°C until the date of analysis. The serum samples were allowed to thaw for up to 30 minutes at room temperature (18–25°C) and inverted three times to ensure sufficient mixture and thawing. Each patient sample was prepared by pipetting 3 μL of serum onto three sample wells of the Dxcover® Sample Slides (Dxcover®, Glasgow, UK). Prepared slides were placed in a drying unit incubator (Thermo Fisher™ Heratherm™, GE) at 35°C for 1 hour, to control the dehydration process of the serum droplets.^35^ The dried sample slides were loaded on to the Dxcover® Autosampler (Dxcover®, Glasgow, UK) and prepared for spectral collection. In this study, a Perkin Elmer Spectrum 2 FTIR spectrometer (Perkin Elmer, USA) was used to generate spectral data (16 co-added scans at 4 cm^–1^ resolution, with 1 cm^–1^ data spacing). A total of three spectra were collected for each sample well, resulting in nine replicates per patient. The 9 patient spectra were fed into the diagnostic algorithm which generates the disease prediction. Anonymized samples were reported as brain cancer positive or negative according to spectroscopy test results.

### Algorithm Training

Following spectral acquisition, the nine spectra obtained from each patient sample were analyzed using the Dxcover® Brain Cancer Algorithm—a machine learning model trained and tuned to accurately detect the signal of brain cancer. This algorithm was trained on 385 prospectively recruited patients,^34^ which were distinct from the 603-patient blind test set (Supplementary Table S1). This model was chosen through a nested cross-validation approach, in which 5-fold cross-validation was used to optimize model hyperparameters and the probability threshold. This was repeated for 51 separate train-test splits to obtain a robust measure of the performance of the model. The model was then retrained on the full 385-patient dataset using the optimal hyperparameters and probability threshold and predictions were made on the analyst-blinded 603-patient cohort, the results of which are presented in this paper.

### Disease Prediction

A consensus prediction was determined from the 9 spectra acquired from each patient. In cases where 5 or more spectra were predicted as positive then a positive result was reported for the patient. Receiver operating characteristic (ROC) curves were generated by altering the probability threshold of the algorithm between cancer and non-cancer and noting the corresponding sensitivity and specificity. In this study, disease predictions were generated for the anonymized cohort of patients and were submitted for review as part of the interim analysis. Throughout this process, analysts and operators were blinded to the true patient disease class and lead clinician Dr. Paul Brennan was responsible for unblinding and reporting test performance against CT. The reference standard in this study was CT imaging, to confirm or refute evidence of central nervous system tumors, followed by diagnosis by biopsy if clinically indicated.

## Results

### Patient Cohort Data

**Figure 2.**
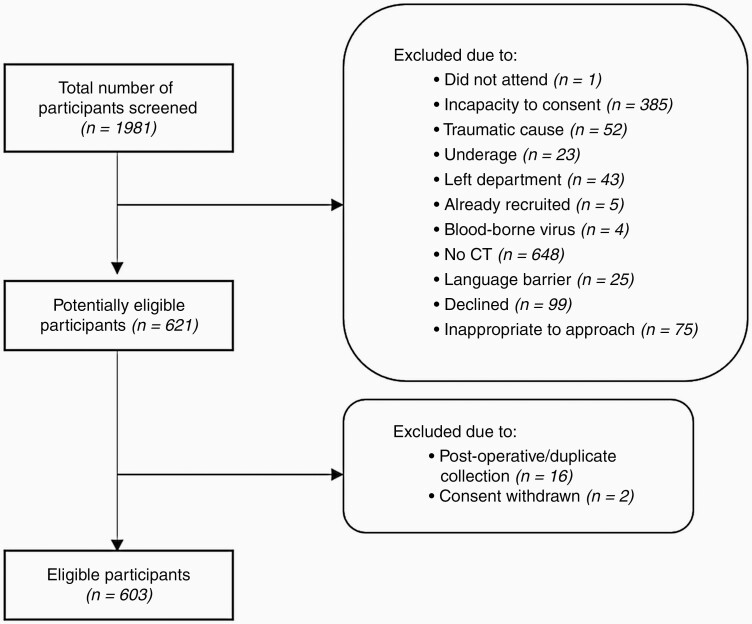
Flow of participants through prospective recruitment for the clinical validation study.

A total of 1981 patients from a diverse, urban population were screened for eligibility based on the primary care referral pathway for brain imaging. The flow of participants is highlighted in Figure 2. The age and sex breakdown of the 603 patients included in this study are presented in the supplementary material (Supplementary Table S2). While we did not stratify based on race/ethnicity, these patients represented a diverse, urban patient population. In total, 47 patients had a confirmed brain tumor, resulting in a prevalence of 7.8%. The most common and malignant brain tumor in adults—glioblastoma (GBM)—was found to be the most prevalent in this population (*n =* 20). There were also 12 brain metastases observed: breast (*n =* 6), lung (*n =* 4), and esophagus (*n =* 1), metastatic tumor of unknown primary (*n =* 1). The remaining tumors were meningioma (*n =* 10), low-grade glioma (*n =* 2), primary central nervous system lymphoma (*n =* 2), and medulloblastoma (*n =* 1). The final diagnosis for the non-cancer patients is also described in the supplementary information (Supplementary Table S3).

**Figure 3.**
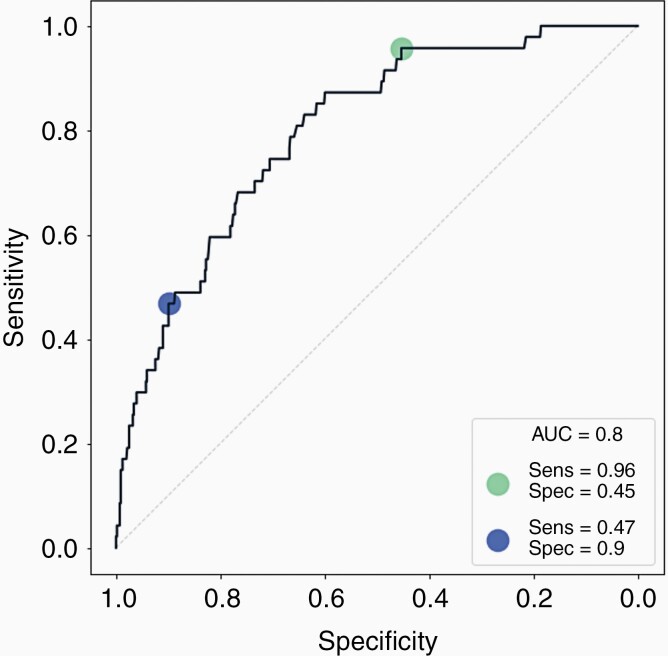
Receiver operating characteristic (ROC) curve, showing the trade-off between sensitivity (Sens) and specificity (Spec) at varying probability thresholds. Blue circle, sensitivity-tuned; Green circle, specificity-tuned. AUC; area under the curve.

Test performance was determined by comparison of the test prediction against the reported diagnosis confirmed via CT brain imaging. Predictions of the clinical data were performed in an analyst-blind fashion before comparison with the clinical data. To analyze this data, the area under the curve (AUC) of the ROC curve is a useful metric to assess the inherent validity of the diagnostic test.^36^ The ROC curve shown in Figure 3. reports an AUC value of 0.8, which indicates the test has an excellent discriminating ability.^37^

Sensitivity or specificity can be maximized depending upon the requirements of the diagnostic pathway and healthcare system. For example, sensitivity (or specificity) can be augmented while ensuring the specificity (or sensitivity) is at an approximate value. If greater sensitivity is required, we can achieve 96% sensitivity (green circle) with a 45% specificity. If a higher specificity is desired (blue circle), a 90% specificity results in a 47% sensitivity. The full breakdown of diagnostic performances for both approaches (sensitivity- and specificity-tuned) in the 603 patient population is summarized in Table 1.

**Table 1.** Summary of Diagnostic Performance for Both the Sensitivity-Tuned and Specificity-Tuned Algorithms

	Sensitivity-tuned	Specificity-tuned
Sensitivity	96%	47%
Specificity	45%	90%
Prevalence	7.8%	7.8%
PPV	12.9%	28.4%
NPV	99.3%	95.3%

PPV, positive predictive value; NPV, negative predictive value.

The predictions by brain tumor type, for the model tailored for greater sensitivity, are shown in Table 2. The detection rates for the specificity-tuned model can be found in Supplementary Table S4. Significantly, the sensitivity-tuned algorithm detected 100% (20/20) of GBM patients correctly, the most common and aggressive adult brain tumor. The Dxcover® Brain Cancer liquid biopsy also predicted 100% of the metastatic (*n* = 12), lymphoma *(n* = 2), and medulloblastoma (*n* = 1) tumors, and 90% of meningiomas (*n* = 10) were detected successfully. Of the two low-grade gliomas in this cohort, one was predicted correctly.

**Table 2.** Detection Rates by Brain Tumor Type for the Sensitivity-Tuned Algorithm

Tumor Type	Actual	Identified	Detection Rate (%)
GBM	20	20	100
Meningioma	10	9	90
Metastatic	12	12	100
Lymphoma	2	2	100
Low grade glioma	2	1	50
Medulloblastoma	1	1	100
**TOTAL**	**47**	**45**	**96**

The spectral regions, or specific wavenumbers, that contribute to a classification can be assessed by feature importance analysis. The wavenumber regions that were found to be most discriminatory for the can be visualized in importance plots (Supplementary Figure S2). The top 5 regions of importance are described in Supplementary Table S5, with tentative biological assignments and their corresponding vibrational modes.^38^

### Patient Metadata

We explored patient metadata was explored to assess any impact on the diagnostic predictions of the brain cancer liquid biopsy test. Supplementary Table S2. outlines the patient metadata breakdown of the 603 patients involved in this study. Upon analysis of demographic and clinical characteristics, no distinct patterns were observed. In particular, patient age did not significantly affect either the sensitivity-tuned or specificity-tuned models (Supplementary Figure S2). The detection rates for both models when split by sex, head pain, and personality change are described in Supplementary Table S6, neither of which indicate any concerns about these potential confounding factors. Since many non-cancer patients in the cohort had a stroke diagnosis, we further assessed whether this would have an effect on the diagnostic predictions. The detection rates for the patients with and without stroke were examined (Supplementary Table S6), and it can be deduced that stroke does not appear to have an influence on the test performance. This shows great promise as it indicates that the brain cancer liquid biopsy is accurate, robust, and dependable in clinical applications where patients may have confounding characteristics and co-morbidities that may result in the presentation of similar non-specific symptoms.

## Discussion

We examined the clinical utility of a spectroscopic-based liquid biopsy on prospectively collected samples from patients with proven or suspected brain cancer. The study was designed to provide a cohort of patients that are as close as possible to the target population of those with non-specific symptoms of head pain and/or neurological deficits, which may be indicative of a brain tumor. Our findings demonstrate the significant impact this rapid, cost-effective test can have in supporting earlier diagnosis of brain tumors.

There were 47 brain tumors diagnosed in this 603-patient cohort. The test performance was effective in predicting GBM, glioma, lymphoma, and metastatic cancers. These are the most common and aggressive types of brain tumors which require rapid instigation of therapy and will benefit most from earlier diagnosis. There is a reduction in the morbidity of brain tumor surgery and radiotherapy when clinicians can diagnose brain tumors at an earlier stage when these tumors may have a significantly smaller volume/mass. When tuned for a greater sensitivity the brain cancer liquid biopsy also identified 9/10 meningiomas. Although meningiomas may not require urgent surgical intervention, detecting these more indolent tumors would give patients and their clinicians invaluable information directly influencing the monitoring, management, and treatment of their condition.

The symptoms most common in brain tumor patients are non-specific and the prevailing diagnostic paradigm, dependent on the clinical history and examination to guide brain imaging referral patterns, is largely ineffective. This is evidenced by the 62% of patients who remain undiagnosed until symptoms force them to appear in the ED,^5^ the excessive number of primary care consultations for each patient before diagnosis, and the very low diagnostic rate from imaging referrals—a recent study showed that for every 60 brain scans only 1 will result in a brain tumor.^39^ Crucially, the Dxcover® Brain Cancer liquid biopsy can significantly enhance this existing cancer referral pathway, supporting expert clinical decision-making. Since this liquid biopsy involves a simple, non-invasive blood test, with a fast turnaround time (24–48 hours or earlier) it can be used in any clinical setting. Incorporating this technology into the primary care setting, particularly in areas where access to specialists and more advanced medical equipment may be limited, could help to reduce health care disparities for vulnerable populations. In addition, incorporating this tool into existing referral protocols could save time, money, and assets by avoiding unnecessary imaging, additional tests/follow-up procedures, and reducing the burden on limited resources and staff. The impact of an early and efficient diagnosis can be the determining factor in these patients’ prognosis and outcome while contributing to considerable savings in costs for patients and healthcare systems.

Current guidelines in the UK to support primary care doctors in identifying patients most at-risk of having a brain tumor include the Kernick referral criteria that utilize a red/orange/yellow flagging system to reflect three levels of risk for brain tumor.^40^ Similarly, the 2005 NICE referral guidelines include details of symptoms that should prompt urgent referral, consideration of urgent referral, or non-urgent referral.^41^ Despite having this guidance in place, it is still extremely difficult for physicians to know whether their patients should be expedited for a brain scan. Thus, the existing referral guidelines are still insufficient in stratifying patients based on their symptoms.^7^ In both orange and yellow flag groups many patients with brain cancer will not be referred for imaging until prompted by costly symptom progression.

A blood test for brain cancer would reduce the proportion of patients being diagnosed in ED as well as the number of unnecessary medical imaging tests performed, thus releasing pressure on the imaging system and saving healthcare costs. Within our previously published health economic studies, we assume half of the patients with a negative result will still undergo imaging.^24,25^ With the lower cost of the blood test compared to imaging, and with up to 50% fewer brain scans, not only would a brain cancer liquid biopsy be economically effective but all patients waiting for imaging would be scanned quicker. In patients presenting to their primary care doctor, where a brain tumor is suspected, a positive blood test result will permit the prioritization of that patient for brain imaging, reducing the risk that they present to the ED with preventable clinical deterioration while awaiting delayed diagnostic brain imaging. Furthermore, a positive result in our liquid biopsy could potentially negate the need for a CT scan, with risk of radiation and imperfect sensitivity for tumors, it may be preferred to proceed directly for an MRI scan. A negative blood test, if consistent with the physician’s clinical assessment of low likelihood of a brain tumor, would enable the avoidance of brain scanning. If clinical suspicion persists despite a negative blood test, further clinical assessment or investigations can be arranged.

A recent study examining the NICE 2005 and Kernick referral guidelines for symptoms suggestive of brain tumor reported a PPV of 2.9% and 2.8% for the ’symptoms related to CNS’ and “red flag symptoms” respectively, the categories of highest perceived risk.^7^ On an equivalent basis, our sensitivity-tuned model indicates a PPV of 12.9%, thus providing more than a 100-fold improvement in detection when compared to headache alone, which has a 0.1% PPV.^42^ This is also greater than the highest published PPV (7.2%) which was associated with a combination of symptoms (headache, cognition, concentration, and confusion symptoms) over a prolonged 6-month period where the disease will be progressing.^42^ Furthermore, the PPV of the Dxcover® Brain Cancer liquid biopsy increases when utilizing the specificity-tuned model, with a reported value of 28.4%. However, a high NPV is imperative for a triage test, particularly for early detection of cancer as it defines the probability that the patients with a negative test result truly do not have the disease. In other words, it is vital to have a low number of false negatives, meaning the negative predictions are indeed true negatives. Our results indicate this would be achievable, with an extremely high NPV of 99.3%.

In a population of symptomatic patients with suspicion of brain cancer who are referred for brain imaging, the actual proportion of brain tumor diagnoses is approximately 1%.^39^ To estimate the impact of the liquid biopsy (Supplementary Table S7), if we assume a 1% prevalence amongst 10 000 symptomatic patients being tested, then 100 would likely have brain tumors.^39^ A recent case-control study reported a sensitivity of 14.2% for headache alone, meaning based on the current pathway only 14 of the 100 brain tumor patients would receive a timely diagnosis.^42^ However, with our sensitivity-tuned algorithm 96 of these patients would receive a positive result and receive an urgent referral to imaging for confirmation of the diagnosis. These patients will obtain a much more timely a diagnosis than current standard care. The other 4 would be referred for imaging only when they present with persistent, additional, or worsening symptoms. Such “safety netting” for symptom progression is a standard part of clinical care. If a physician remains concerned about a possible cancer diagnosis despite a negative liquid biopsy test, brain imaging could still be arranged. In addition, 38% of brain tumor patients visit their doctor more than five times prior to diagnosis. Even if the liquid biopsy test did not identify a patient’s tumor initially, a repeated blood test at a subsequent visit could still enable quicker referral saving precious diagnostic and therapeutic time and resources. For the sensitivity-tuned model, 4455 of the 9900 patients without a brain tumor would receive a negative result and have a routine follow-up. The 5445 patients who received false positives would be fast-tracked for imaging. It is likely these patients would have been offered a brain scan eventually based on the current pathway, yet a quicker referral would allow clinicians to rule out a brain tumor earlier. In particular, this technology would be useful to patients with an inherited predisposition or those requiring surveillance. A simple blood test that can detect tumors early and easily could be a beneficial and cost-effective part of the standard screening protocols for these patients.

With our specificity-tuned model, 47 out of the 100 brain tumor patients will receive a positive result and get an urgent referral for imaging. Among the 9900 patients without a brain tumor, 8910 would receive a negative result with our specificity-tuned algorithm. Thus, with fewer false-positive tests than the sensitivity tuned model the cost of imaging would be markedly lower, with fewer incidental findings anticipated. The 53 brain tumor patients who received a false negative result but have clinical symptoms that fall within standard risk guidelines, would still meet eligibility requirements to obtain a diagnosis along the standard diagnostic pathway, either because the physician refers them directly for brain imaging as they remain concerned about the patient, or because the patient later presents with persistent or progressive symptoms. A repeated blood test at a follow-up visit could still enable quicker referral. In either case, the time to diagnosis will have been substantially decreased for 47% of patients, potentially unchanged for 53% of patients, and the overall referral for imaging markedly reduced.

In our 10 000 (1% prevalence) population, with a single computed tomography (CT) brain scan costing approximately £90 in the UK, the savings for the NHS could be substantial.^24^ With the specificity-tuned algorithm we would significantly reduce unnecessary medical imaging on these patients. As well as CT, many patients in the diagnostic pathway will undergo magnetic resonance imaging (MRI) in the UK, which is more expensive and costs around ~£165 per scan.^25^ In the US, these costs can be much higher and can vary drastically depending on many factors including whether or not the patient has health insurance, the clinician or hospital is within the network, the particular equipment available, the clinical setting where the procedure is performed, and the geographical location among other factors. Costs for MRIs for example, can range from $375 to $2850 with the US national average cost around $1325, while costs for brain CT scans can range from $825 to $4800 with the national average cost around $1200.^43,44^Supplementary Table S8 overviews the potential cost savings with this scenario in the UK and US. For the sensitivity-tuned model, it is estimated that the UK’s NHS could save around £1 136 025 per 10 000 patients. Likewise, with scans being more expensive in the US, approximately $11 248 875 could be saved with the use of the sensitivity-tuned model. Utilization of the specificity-tuned model would enable even greater cost savings, with ~£2 272 050 in the UK and $22 497 750 in the US. It is worth noting that the estimates presented here are solely based on the costs of brain scans, and do not consider the additional expenditures for neuropathology outpatient appointments and numerous primary care visits, which with the addition of the liquid biopsy could be avoided for a substantial proportion of patients. The precise trade-off between sensitivity and specificity may be specific to different regional and national healthcare systems and clinician discretion. From either perspective, a brain cancer liquid biopsy reduces the total number of brain imaging investigations needed, decreasing time to diagnosis for patients with a positive blood test and an actual tumor, as well as significantly reducing overall healthcare costs and resources.^24,25^

There are no currently available triage tests for brain cancer, but a commercial comparator for clinical benchmarking can be provided by the Select MDx test for prostate cancer (MDx Health).^18^ In a similar fashion to the use of the Dxcover® Brain Cancer liquid biopsy, this test is directed at symptomatic patients who have an abnormal prostate-specific antigen (PSA) level or abnormal digital rectal exam (DRE). The results from the Select MDx genomic urine liquid biopsy are used to decide if the patient moves forward with medical imaging followed by biopsy or just to routine PSA follow-up.^45^ Another potential comparator is the ExoDx test, commercialized by the company ExosomeDx.^19^ The ExoDx test is a urine-based liquid biopsy that provides a risk score to determine a patient’s risk of clinically significant prostate cancer on a prostate biopsy.^46^ When comparing the diagnostic performance of these commercial tests against the results from our 603-patient dataset, the Dxcover® Brain Cancer liquid biopsy sensitivity-tuned model performs favorably (Table 3), highlighting that our simple blood test can be as effective as commercially available tests for cancer.

**Table 3.** Comparison of the Diagnostic Performance of the Sensitivity-Tuned Model Against Commercially Available Liquid Biopsies for Cancer^44,45^

Liquid Biopsy	Targeted Cancer	Patient Cohort	Sensitivity (%)	Specificity (%)	Prevalence (%)	NPV (%)	AUC
Dxcover® Brain Cancer (Dxcover Ltd.)	Brain	603	96	45	7.8	99.3	0.8
Select MDx (MDx Health)	Prostate	916	93	47	26	95	0.85
ExoDx (Exosome Dx)	Prostate	503	90	38.6	32	89.3	0.71

NPV, Negative predictive value; AUC, area under the curve.

The optimum balance of sensitivity and specificity will be informed by the specific healthcare system in which the test is being applied and the discretion of the physician using the test. A reduction in specificity increases the false-positive results which will likely result in more brain imaging, whilst a reduction in sensitivity will increase the possibility that a patient with a tumor will be initially missed. There is arguably the most value to be gained in detecting some, but not all, brain tumors earlier. Significantly more false-positive results would ultimately lead to increased number of referrals for brain imaging, which may be less welcome. Hence the optimum balance may favor specificity more than sensitivity. In addition, the barrier for access to a blood test may be substantially lower than the barrier for access to more costly medical imaging tests, meaning more blood tests can be easily conducted. The integration of a brain cancer liquid biopsy into existing pathways would permit more effective triage of patients, expediting assessment for those most at-risk whilst excluding a brain tumor diagnosis in others. By decreasing the time to diagnosis, the morbidity from treatment can be reduced, which in turn would improve the quality of life of brain cancer patients, resulting in a greater prognosis.

## Supplementary Material

vdac024_suppl_Supplementary_Material

## Data Availability

The datasets analyzed within the scope of the study cannot be published publicly due to privacy regulations under the General Data Protection Regulation (EU) 2016/679. The raw data includes clinical data from patients, including textual clinical notes and contains information that could potentially compromise subjects’ privacy or consent.
