# Supplementary material for: Clinical validation of a spectroscopic liquid biopsy for earlier detection of brain cancer
Source: Neurooncol Adv. 2022 Feb 22;4(1):vdac024. doi: 10.1093/noajnl/vdac024 (PMC8934542; doi:10.1093/noajnl/vdac024)
Supplement: vdac024_suppl_Supplementary_Material [file vdac024_suppl_supplementary_material.docx]

Clinical validation of a spectroscopic liquid biopsy for early detection of brain cancer

**Authors:** James M. Cameron^1†^, Paul M. Brennan^2†^, Georgios Antoniou^1^, Holly J. Butler^1^, Loren Christie^1^, Justin J.A. Conn^1^, Tom Curran^3^, Ewan Gray^4^, Mark G Hegarty^1^, Michael D. Jenkinson^5^, David S. Palmer^1^, Daniel Orringer^6^, Alexandra Sala^1,7^, Benjamin R. Smith^1^, Matthew J. Baker^1^*

**Supplementary Materials**
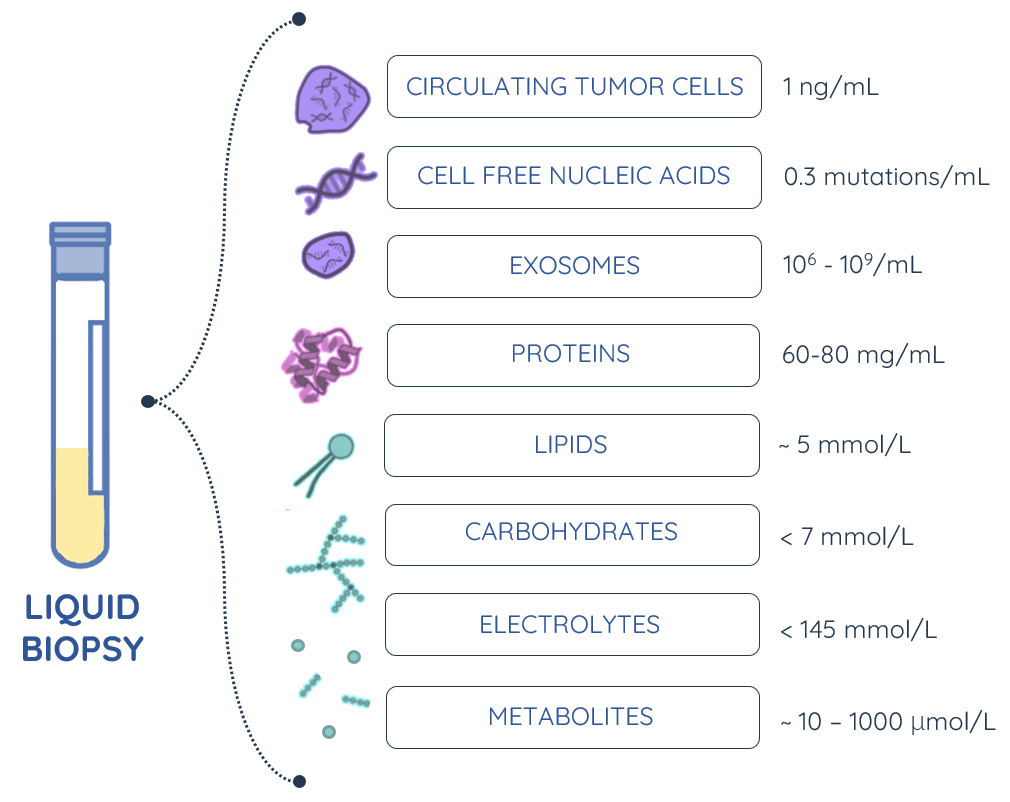


**Fig. S1. Schematic showing the range of biomarkers that are contained within a human blood sample.**

**Table S1.** Age and sex information about the prospectively recruited 385-patient cohort that was used to train the diagnostic algorithm *^34^.*

|  | | | |
| --- | --- | --- | --- |
|  |  | **Cancer (M/F)** | **Non-cancer (M/F)** |
| **Age** | 20+ | 0 / 0 | 5 / 14 |
|  | 30+ | 2 / 3 | 16 / 20 |
|  | 40+ | 4 / 5 | 12 / 18 |
|  | 50+ | 2 / 6 | 23 / 28 |
|  | 60+ | 12 / 9 | 20 / 44 |
|  | 70+ | 8 / 5 | 35 / 36 |
|  | 80+ | 4 / 5 | 23 / 21 |
|  | 90+ | 0 / 1 | 3 / 1 |
| **Total** |  | 66 | 319 |

**Table S2.** **Patient metadata for the 603-dataset included in this study.** Where data was unavailable or not recorded, ‘NA’ (Not available) was noted.

| **Metadata** | | **Cancer** | **Non-cancer** |
| --- | --- | --- | --- |
| **Sex** | F | 24 | 253 |
|  | M | 23 | 303 |
| **Age decile** | 10+ | 0 | 2 |
|  | 20+ | 0 | 32 |
|  | 30+ | 3 | 48 |
|  | 40+ | 6 | 65 |
|  | 50+ | 11 | 107 |
|  | 60+ | 13 | 120 |
|  | 70+ | 10 | 104 |
|  | 80+ | 4 | 68 |
|  | 90+ | 0 | 10 |
| **Referral Reason Memory Disturbance** | No | 16 | 543 |
|  | Yes | 0 | 9 |
|  | NA | 31 | 4 |
| **Referral Reason Personality Change** | No | 16 | 551 |
|  | Yes | 2 | 1 |
|  | NA | 29 | 4 |
| **Referral Reason Seizure** | No | 9 | 512 |
|  | Yes | 17 | 40 |
|  | NA | 21 | 4 |
| **Referral Reason Focal Weakness** | No | 13 | 412 |
|  | Yes | 8 | 140 |
|  | NA | 26 | 4 |
| **Referral Reason Speech Problem** | No | 14 | 491 |
|  | Yes | 9 | 61 |
|  | NA | 24 | 4 |
| **Referral Reason Visual Problem** | No | 15 | 521 |
|  | Yes | 3 | 32 |
|  | NA | 29 | 3 |
| **Referral Reason Vomiting** | No | 16 | 544 |
|  | Yes | 1 | 7 |
|  | NA | 30 | 5 |
| **Referral Reason Confusion** | No | 15 | 530 |
|  | Yes | 5 | 21 |
|  | NA | 27 | 5 |
| **Referral Reason Sensory** | No | 15 | 539 |
|  | Yes | 0 | 13 |
|  | NA | 32 | 4 |
| **Referral Reason Collapse** | No | 15 | 524 |
|  | Yes | 0 | 28 |
|  | NA | 32 | 4 |
| **Referral Reason Dizziness** | No | 15 | 473 |
|  | Yes | 3 | 79 |
|  | NA | 29 | 4 |
| **Referral Reason Coordination** | No | 14 | 540 |
|  | Yes | 2 | 12 |
|  | NA | 31 | 4 |
| **Head pain** | No | 27 | 284 |
|  | Yes | 20 | 271 |
|  | NA | 0 | 1 |
| **Headache Duration** | Hours | 2 | 92 |
|  | Days | 3 | 117 |
|  | Weeks | 7 | 71 |
|  | NA | 35 | 276 |
| **Headache Worse in morning** | No | 9 | 72 |
|  | Yes | 4 | 211 |
|  | NA | 34 | 273 |
| **Headache with Nausea** | No | 11 | 153 |
|  | Yes | 2 | 131 |
|  | NA | 34 | 272 |
| **Memory Change** | No | 35 | 143 |
|  | Yes | 9 | 412 |
|  | NA | 3 | 1 |
| **Personality Change** | No | 30 | 74 |
|  | Yes | 17 | 481 |
|  | NA | 0 | 1 |
| **Stroke** | No | 47 | 317 |
|  | Yes | 0 | 239 |
| **Verbal Fluency** | 15 + | 16 | 370 |
|  | < 15 | 7 | 185 |
|  | NA | 24 | 1 |

**Table S3. Final diagnosis of non-brain tumor patients.**

| **Final Diagnosis** | **Number of patients** |
| --- | --- |
| Ischaemic stroke | 53 |
| Chronic subdural haematoma | 8 |
| Subarachnoid haemorrhage | 3 |
| Haemorrhagic stroke | 3 |
| Venous sinus thrombosis | 3 |
| Longstanding epidermoid residuum | 1 |
| Incidental aneurysm | 1 |
| Neurocysticercosis | 1 |
| Cavernoma | 1 |
| No significant abnormality | 467 |
| Final diagnosis not recorded | 15 |

**Table S4.** **Detection rates by brain tumour type for the specificity-tuned algorithm.**

| **Tumour Type** | **Actual** | **Identified** | **Detection Rate (%)** |
| --- | --- | --- | --- |
| GBM | 20 | 11 | 55 |
| Meningioma | 10 | 3 | 30 |
| Metastatic | 12 | 5 | 42 |
| Lymphoma | 2 | 2 | 100 |
| Low grade glioma | 2 | 0 | 0 |
| Medulloblastoma | 1 | 1 | 100 |
| **TOTAL** | **47** | **22** | **47** |

**
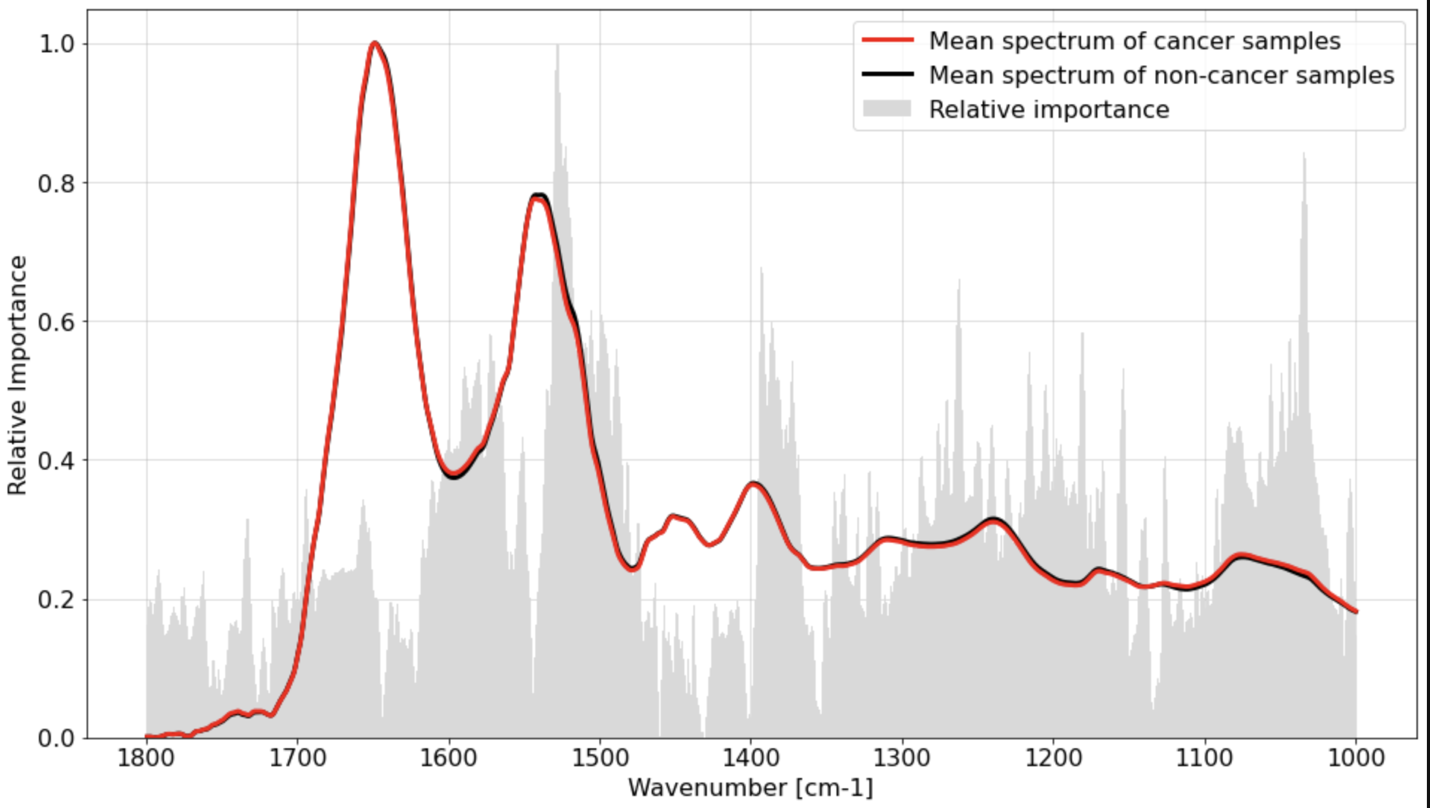
**

**Fig. S2. Feature importance plot highlighting the wavenumber regions that were found to be the most discriminatory for the diagnostic predictions.** The importance values were normalized to a maximum of 1 to make them more comparable.

**Table S5. The top 5 wavenumber regions which were found to be the most discriminatory for diagnostic predictions, with their corresponding tentative biological assignments and vibrational modes.**

| **Approximate Wavenumber (cm^-1^)** | **Biological Assignments** | **Vibrational Modes** |
| --- | --- | --- |
| 1530 | Amide II of Proteins | N-H bending, C-N stretching |
| 1035 | Glycogen, Carbohydrates | C-O and C-C stretch,  C-OH deformation |
| 1393 | Lipids, Proteins | C-O stretch, C-H and N-H deformation |
| 1260 | Amide III of Proteins, Phosphodiesters | N-H in-plane bend, C-N stretch, asymmetric P$O_{2}^{-}$stretch |
| 1180 | Amide III of Proteins,  Nucleic Acids | N-H in-plane bend, C-N stretch, asymmetric P$O_{2}^{-}$stretch |


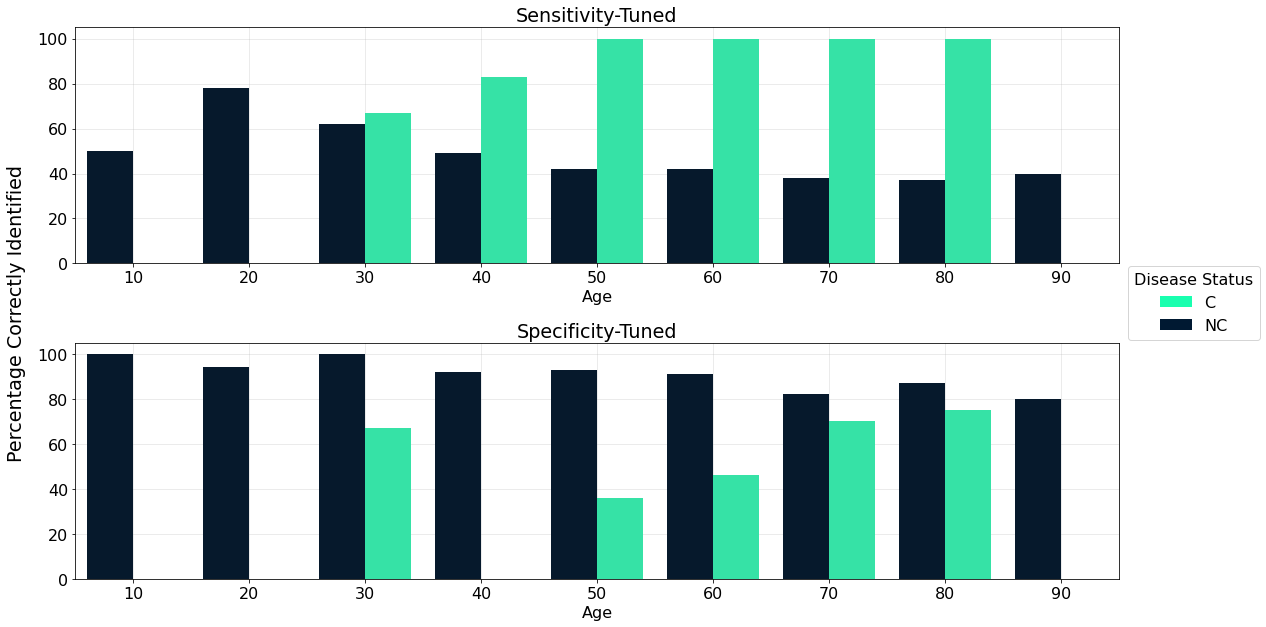


**Fig. S3**. **Patient age distribution across prediction accuracy for cancer (green) and non-cancer (blue).** Age was collected in discrete decile groups for patient anonymity.

**Table S6. Detection rates for the sensitivity and specificity-tuned models split by metadata factors.** NA is noted as no cancer patients were found to have stroke.

| Metadata | **Detection rate (%)** | | | | | | | |
| --- | --- | --- | --- | --- | --- | --- | --- | --- |
|  | Sensitivity-tuned | | | | Specificity-tuned | | | |
|  | C | | NC | | C | | NC | |
|  | Male/Yes | Female/No | Male/Yes | Female/No | Male/Yes | Female/No | Male/Yes | Female/No |
| Sex (Male/Female) | 100 | 92 | 47 | 44 | 57 | 38 | 89 | 91 |
| Head pain (Yes/No) | 95 | 96 | 39 | 51 | 35 | 56 | 87 | 93 |
| Stroke (Yes/No) | NA | 96 | 42 | 48 | NA | 47 | 89 | 91 |
| Personality Change (Yes/No) | 94 | 97 | 47 | 38 | 24 | 60 | 90 | 92 |

**Table S7.** Clinical scenarios of Dxcover® Brain Cancer liquid biopsy applied to primary care referral population of 10,000 patients.

|  | Test result and outcome | Sensitivity-tuned | Specificity-tuned | Patient impact |
| --- | --- | --- | --- | --- |
| *Patients with brain cancer (n=100)* | Test positive emergency referral | 96 | 47 | Diagnosed earlier than standard care |
|  | Test negative no further testing | 4 | 53 | Diagnosed at the same time as standard care |
| *Patients without brain cancer (n=9,900)* | Test positive emergency referral | 5,445 | 990 | Experience more urgent imaging than standard care |
|  | Test negative no further testing | 4,455 | 8,910 | Avoid unnecessary imaging |

**Table S8.** **Potential cost savings with the implementation of the brain cancer liquid biopsy in the UK and US.** Estimates in a population of 10,000 symptomatic patients being tested, where computed tomography (CT) and magnetic resonance imaging (MRI) scans in the UK are ~£90 and £165, respectively, and the national average cost in the US of $1,200 for CT and $1,325 for MRI ^24, 25, 42, 43^.

|  | UK | | | US | | |
| --- | --- | --- | --- | --- | --- | --- |
|  | CT | MRI | Total | CT | MRI | Total |
| *Sensitivity-tuned* | £400,950 | £735,075 | **£1,136,025** | $5,346,000 | $5,902,875 | **$11,248,875** |
| *Specificity-tuned* | £801,900 | £1,470,150 | **£2,272,050** | $10,692,000 | $11,805,750 | **$22,497,750** |
